# Protective Effects of Metformin on Renal Tubular Cells

**DOI:** 10.5812/ircmj.11662

**Published:** 2014-09-05

**Authors:** Mahmoud Rafieian Kopaei, Azar Baradaran

**Affiliations:** 1 Medical Plants Research Center, Shahrekord University of Medical Sciences, Shahrekord, IR Iran; 2 Department of Pathology, Isfahan University of Medical Sciences, Isfahan, IR Iran

**Keywords:** Metformin, Kidney, Toxicity

*Dear Editor,*

Recently, we conducted a preclinical investigation to find the ameliorative properties of metformin on renal biochemical and histologic alterations of gentamicin-induced kidney damage in male Wistar rats (1). In this investigation, attenuation of gentamicin-induced acute kidney injury was found. Likewise, Taheri et al. conducted a study on the effects of metformin on renal tubular cells after unilateral ischemia reperfusion in rats’ kidney. They observed that metformin provided kidney protection against ischemia and reperfusion-induced injury (2). They concluded that metformin has tissue-protective effect through activation of adenosine monophosphate-activated protein kinase (AMPK) and endothelial nitric oxide synthase (2). More recently we conducted a study on 70 male Wistar rats to test the efficacy of coadministrating garlic extract and metformin in prevention of gentamicin-induced renal tubular damage in Wistar rats (3). The result of this study showed that metformin, garlic juice, or their combination had both curative and protective effects on gentamicin-induced kidney injury. In addition, Kim et al. conducted a study using metformin for diabetic rats for 17 weeks and found that treatment of diabetic rats with metformin had restored podocyte loss. They suggested that diabetes-induced podocyte loss in diabetic nephropathy could be suppressed by metformin through the repression of oxidative injury. They proposed that diabetes-induced podocyte loss in diabetic nephropathy could be reduced by metformin (4). Kim et al. found that the phosphorylation of adenosine monophosphate-activated protein kinase (AMPK) was decreased in the kidney of diabetic rats and metformin could restore its modification (4). Diabetic nephropathy is one of the most important complications of diabetes mellitus (5-11) and metformin has been broadly used for the treatment of type 2 diabetes (12). Thus, the suggestion of Baradaran et al. further attests our results and those by Taheri et al., which stated metformin protects against tubular injury by restoring the biochemical alterations and modulation of oxidative stress on the tubules (2, 3). Furthermore, according to the study of Kim et al. metformin protects podocytes in diabetic nephropathy (4). On the other hand, there is also tubular cell injury in diabetic nephropathy due to glycosuria (12-18). These findings can more potentiate the clinical use of metformin in the prevention of diabetic nephropathy. Previously, Morales et al. showed that gentamicin-induced renal tubular damage was attenuated by metformin (17). It is evident that metformin treatment significantly attenuates the increase in malondialdehyde and total reactive oxygen species generation and restores both enzymatic and nonenzymatic antioxidants to their physiologic levels (18, 19). These findings advocate the use of metformin in diabetes due to its protective effect on kidney beyond its blood regulatory effects. In fact, it is reasonable to illuminate three different actions of metformin including blood sugar regulatory property, renal tubular cell protection by acting as an effective antioxidant, and finally, protective effect on diabetic nephropathy through saving the podocytes (4, 18, 19). Hence, patients with diabetes might benefit from all of these three distinct protective properties (4, 18, 19). In this regard, more experimental or clinical studies are recommended to improve our knowledge regarding the kidney protective properties of metformin.
